# Evaluation of Mini-Mental State Examination scores according to different age and education strata, and sex, in a large Brazilian healthy sample

**DOI:** 10.1590/S1980-57642009DN30200004

**Published:** 2009

**Authors:** Renata Kochhann, Maria Otília Cerveira, Cláudia Godinho, Analuiza Camozzato, Márcia Lorena F. Chaves

**Affiliations:** 1,2 MD, Dementia Clinic, Neurology Service, Hospital de Clínicas de Porto Alegre. Medical Sciences Post-Graduate Course, UFRGS School of Medicine, Porto Alegre, RS, Brazil.; 3 MD, PhD, Dementia Clinic, Neurology Service, Hospital de Clínicas de Porto Alegre, Porto Alegre, RS, Brazil.; 4 MD, PhD, Internal Medicine Department, UFRGS School of Medicine, Porto Alegre, RS, Brazil.

**Keywords:** Mini-Mental State Examination, cognition, cognitive assessment, educational attainment, age, sex

## Abstract

Until better measures have been accepted for wider use, the Mini-Mental State Examination (MMSE) will continue to be utilized. In this context, knowledge on characteristics and determinants of its distribution for the Brazilian population are particularly valuable. The present study aimed to evaluate, based on multivariate analysis, the independent effect of age, educational level and sex, and their interactions, on MMSE scores in a healthy sample.

**Methods:**

Demographic data and scores on the MMSE of 1,553 healthy individuals were analyzed. The sample was grouped according to age and education.

**Results:**

The sample was composed of 963 females (62%), mean age ±SD was 49.6±20.7yrs (range 20 to 92 yrs). The mean years of education ±SD was 8.9±5.5yrs (range 0 to 28 yrs). The mean score ±SD on the MMSE was 27.3±2.7(range 15 to 30). A significant effect of the interaction between education and sex (p=0.011), and also between education and age was observed (p=0.003). An independent effect of education (p<0.001) and age (p<0.001) was found. Participants from the higher educated group presented higher MMSE scores than the other groups. Younger adults presented higher MMSE scores than the other age groups.

**Conclusions:**

We observed an effect of education and age on MMSE scores. Younger individuals and higher educated participants presented higher scores.

## Abstract (pt)

O Mini-Exame do Estado Mental (MEEM) continuará sendo amplamente utilizado até que outras medidas sejam estabelecidas, por isso o conhecimento de características e determinantes de sua distribuição na população é particularmente útil. O presente estudo tem como objetivo avaliar o efeito independente e suas interações da idade, sexo e nível educacional em uma análise multivariada sobre os escores do MEEM em uma amostra saudável.

**Métodos:**

Dados demográficos e escores do MEEM de 1.553 indivíduos saudáveis foram analisados. A amostra foi agrupada de acordo com a idade e a educação.

**Resultados:**

A amostra foi composta de 963 mulheres (62%), a média ±DP da idade foi 49,6±20,7 (variando entre 20 e 92 anos). A média ±DP dos anos de estudo foi 8,9±5,5 (variando entre 0 e 28 anos de estudo). A média ±DP dos escores do MEEM foi 27,3±2,7 (variando entre 15 e 30). Um efeito significativo na interação entre educação e sexo foi observado (p=0,011). Um efeito significativo na interação entre educação e idade também foi observado (p=0,003). Um efeito independente da educação (p<0,001) e da idade (p<0,001) foi observado. Participantes do grupo com alto nível educacional apresentaram maiores escores no MEEM do que os participantes dos outros grupos. Do mesmo modo, adultos jovens apresentaram maiores escores no MEEM do que os participantes dos outros grupos etários.

**Conclusões:**

Nós observamos efeito da educação e da idade sobre os escores do MEEM. Indivíduos mais jovens e indivíduos com alto nível educacional apresentaram escores maiores no MEEM.

The Mini-Mental State Examination (MMSE) was initially developed to screen dementia; however, it has been widely used as a measure of general cognitive functioning. The MMSE is the most widely used screening tool to assess mental or cognitive status in the elderly. Recently, the Brazilian Academy of Neurology^1^ and the American Academy of Neurology^2^ recommended the MMSE as a general cognitive screening instrument for the detection of dementia in individuals with suspected cognitive impairment. In Brazil, various cut-offs points have already been proposed for different educational levels with no consensus reached. However, efforts have been made to standardize its use.^3^ The high numbers of individuals with low levels of literacy yet high levels of illiteracy in some areas of the country, have made estimating the impact of schooling on MMSE scores very important, especially amid reports of reduced specificity^4,5^ among individuals with lower levels of education.^6^

Many studies have demonstrated the effect of age and education on MMSE scores^7-12^ (criterion validity), but education did not show any effect on construct validity.^13^ A longitudinal investigation carried out in healthy elders has shown lower MMSE performance in cognitively impaired elderly particularly among older elderly and the lower educated.^14^ A large multicentric study in the USA showed the same influence of age and education on MMSE scores.^15^ In Brazil, the first evaluation of the impact of education was carried out in 530 subjects aged between 15 and 65 years. Significant differences among groups with different educational levels were observed but did not reach significance for age.^16^ The majority of other Brazilian studies have involved elderly individuals.^17-21^

Longitudinal and cross-sectional studies have shown an age effect on MMSE scores, with the latter having demonstrated stronger associations.^22-24^ Nevertheless, questions remains over whether age-related cognitive decline was normal^24^ or pathological.^25^

Gender differences in cognitive status have also been the subject of investigation with controversial results, in addition to uncertainty as to their correspondent mechanisms. Aspects related to lower female education,^26^ as well as biological differences such as atherosclerosis^27-29^ and hormonal profile^30^ may be involved. Studies have shown higher cognitive performance among women, independent of their lower education,^31^ while no such difference between men and women was observed among older participants.^32^ The applicability of the instruments of cognitive evaluation in different cultures and the impact of different variables such as education, age, and gender on results needs further assessment and standardizing.

In the present study we selected a large sample containing individuals of different ages, including younger participants, and education strata to evaluate the influence of these wide spectra on MMSE performance. The present study aimed to evaluate, based on multivariate analysis, the independent effect of age, educational level and sex, and their interactions, on MMSE scores in a healthy sample.

## Methods

Healthy participants were randomly selected from different sectors of the Hospital de Clinicas de Porto Alegre (Porto Alegre, RS) (relatives, caregivers and visitors) to give a total sample of 1553 participants. Subjects were fully independent, non-demented, and aged from 20 to 92 years. Inclusion criteria were to be functionally independent and cognitively normal. Exclusion criteria were presence of any psychiatric or neurological disease and use of psychoactive drugs. To minimize inclusion of participants with incipient dementia among those aged ≥60, subjects were screened with the Clinical Dementia Rating scale.^33,34^ All participants were tested for hearing^35^ and vision^36^ functions using brief screening tests (the whispered voice test for hearing and the self-reported measure for vision impairment).

Age was classified into different strata: younger adults (20 to 40 years), middle age (41 to 65 years), and older adults (≥66 years). Distribution of education was analyzed in the whole sample to obtain the best categorization. The groups were classified as 0 to 5 years (low education), 6 to 11 years (medium education) and ≥12 years of education (high education). Initially illiterates were placed in separate group, however, since analysis of MMSE performance in the 0 to 5 years of education group revealed no statistical difference (p=0.09) (Bonferroni *post-hoc* test) these years of education were grouped together.

All participants were administered the Mini-Mental State Examination following the same protocol.^37-38^ The MMSE was the main outcome of the study (dependent variable). Age and education strata and sex were the independent factors.

The study was approved by the Ethics Committee for Medical Research at the Hospital de Clinicas de Porto Alegre. All subjects signed an informed consent before being enrolled onto the study.

## Data analysis

Descriptive statistics (mean, SD, and relative frequency) were calculated for demographic data and the MMSE. A univariate general linear model (3-way ANOVA) was designed for the evaluation of the effects of age (young adults, middle age, older adults), education (low, medium, high), gender (male/female), and their interactions on MMSE scores, using the Bonferroni *post-hoc* test. Student’s t test was used for comparing parametric data, and chi-square test for categorical data. For categorization of education, the Bonferroni test was employed to compare MMSE scores by years of schooling. The statistical analysis was performed using the *Statistical Package for the Social Sciences* for Windows (SPSS 13).

## Results

Age ranged from 20 to 92 years, education from 0 to 28 years and MMSE scores from 15 to 30. The sample was grouped according to age and education. The demographic data of the sample is presented in Table 1.

**Table 1:** Distribution of participants according to age and educational level.

Variable	Sample (n=1553)
Age (mean±SD)	49.6±20.7
Age categories (n,%)	
Younger adults	559 (36%)
Middle age	565 (36%)
Older adults	429 (28%)
Education (mean±SD)	8.9±5.5
Categories of education (n,%)	
Low	527 (34%)
Medium	430 (28%)
High	598 (38%)
Gender	
Female (n, %)	963 (62%)
MMSE (mean±SD)	27.3±2.7

A significant effect of the interaction between education and sex was observed (p=0.011) (Table 2). Women from the low education group presented lower MMSE scores than men in low and high education groups. A significant effect of the interaction between education and age was also observed (p=0.003). Older participants from low education groups showed lower MMSE scores. An independent effect of education (p<0.001) and age (p<0.001) was observed. Participants from the high educated group presented higher MMSE scores than the other groups (Bonferroni *post-hoc* test). Younger adults presented higher MMSE scores than the other age groups (Bonferroni *post-hoc* test). Sex did not present an independent effect (not shown in Table 2). No interaction between age and sex was observed.

**Table 2:** Mini-Mental State Examination (mean±SD) in General Linear Model analysis (3way ANOVA): effect of age, education and sex (univariate model).

Effect	MMSE Mean±SD	P value
Age		<0.001
Younger adults (20-40 years)		
Middle age (41-65 years)		
Older adults (≥66 years)		
Education		<0.001
Low (0-5 years)	26.2±0.15	
Medium (6-11 years)	27.2±0.12	
High (≥12 years)	28.1±0.12	
Education* age		0.003
Low		
Younger adults	26.9±0.40	
Middle age	26.0±0.16	
Older adults	25.7±0.17	
Medium		
Younger adults	27.1±0.24	
Middle age	27.7±0.19	
Older adults	27.0±0.23	
High		
Younger adults	28.8±0.12	
Middle age	27.9±0.25	
Older adults	27.5±0.25	
Education* sex		0.011
Low		
Male	26.6±0.26	
Female	25.8±0.16	
Medium		
Male	27.1±0.20	
Female	27.4±0.15	
High		
Male	28.2±0.19	
Female	27.9±0.16	

**Figure 1:**
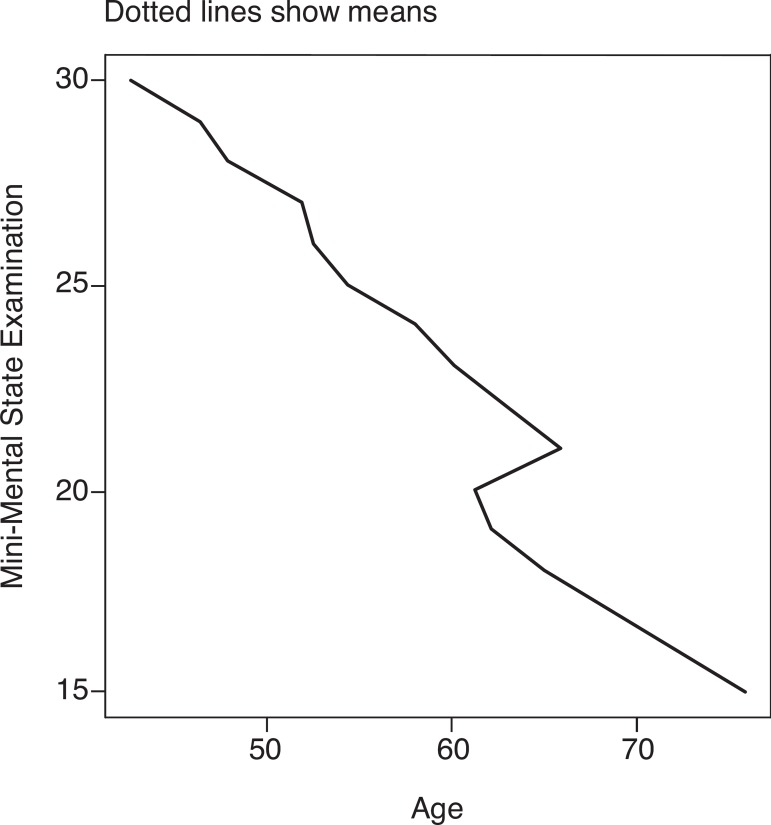
Distribution of Mini-Mental State Examination scores by age (n=1553).

**Figure 2:**
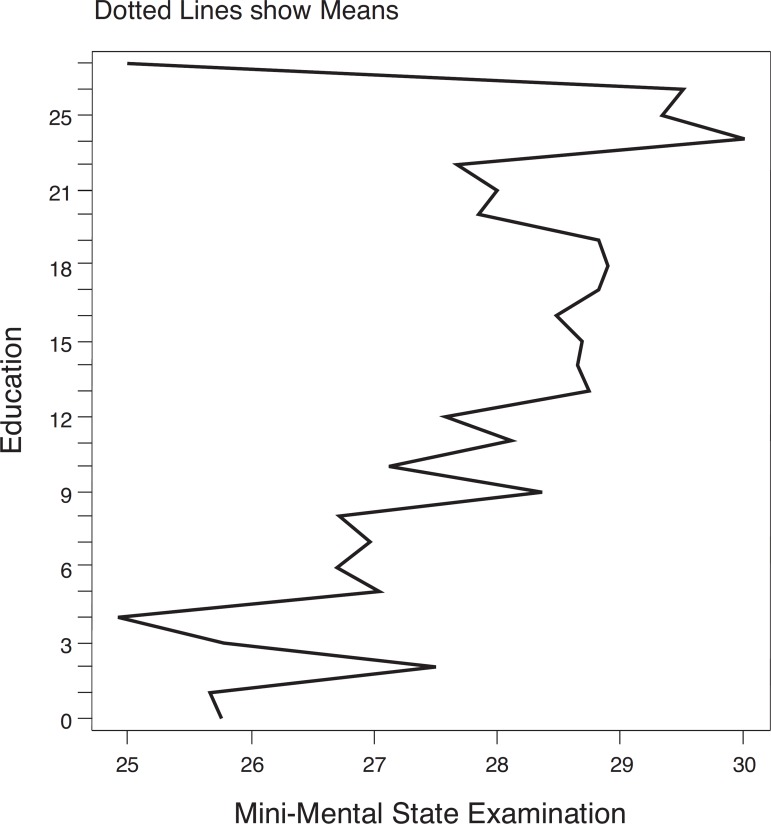
Distribution of Mini-Mental State Examination scores by education (n=1553).

MMSE distribution according to age and education are displayed on Figures 1 and 2.

## Discussion

This study was carried out to evaluate the effect of age, education and gender on MMSE scores in healthy participants. The interaction of education and sex presented a significant effect on MMSE scores, as did the interaction between education and age. Age and education independently influenced MMSE scores, while sex alone did not affect this test.

The influence of education on cognitive performance has been demonstrated in other investigations.^12,39-41^ However, the findings of the present study – education, age, gender and the interactions effects – although not new, are important because they represent complementary knowledge to previous evaluations carried out in Brazil. We assessed all these effects and their interactions in a larger sample of healthy participants. It is also important to highlight the education effect, because it was not exactly linear. There was no significant difference in MMSE scores between illiterates and participants with 0 to 5 years of education. The difference was seen only in comparisons of participants with 6 years of schooling or more, suggesting that individuals without formal education (illiterates) as well as those with lower levels of education may present similar patterns on this mental status screening test. This finding differs to results of earlier studies carried out in other regions of Brazil.^15,19^

In Brazil, primary education is very heterogeneous, with regional characteristics, different yearly and daytime durations, and frequency of teachers.^37^ These differences tend to interfere in research evaluating cognitive performance. Sociological studies and educational evaluations have shown that educational systems reflect social inequalities causing different levels of learning attainment for the same number of years of schooling.^42^

The present study demonstrated a decline in MMSE performance among healthy individuals with age, reinforcing the notion that mental and cognitive status changes with aging may be unrelated to dementia or educational attainment. By taking into account cognitive status change as a normal aging finding, two other characteristics should be carefully considered for the diagnosis of dementia, functional status and intra-individual assessment.

Gender did not affect mental status. Results from studies on this association remain controversial.^31,32^ The cognitive difference observed between sexes has been partially attributed to differential education of men and women (especially among older people), as well as to biological aspects. However, we observed no gender effect on MMSE scores.

Limitations of this study include the greater number of women in the sample, and the fact that participants were healthy – having been selected by excluding medical and psychiatric disorders – which restrict the results to individuals with a similar profile. On the other hand, the strength of the study is in its large sample of healthy participants which minimized the effect of other factors interfering with cognition. Finally, this study offered a rare opportunity to investigate MMSE scores in a large sample of individuals that presented a wide age and education range and who were deemed healthy with respect to conditions affecting cognitive performance.
